# Should Cone-Beam Computed Tomography Be Performed Prior to Orthodontic Miniscrew Placement in the Infrazygomatic Crest Area?—A Systematic Review

**DOI:** 10.3390/biomedicines11092389

**Published:** 2023-08-26

**Authors:** Marcin Stasiak, Paulina Adamska

**Affiliations:** 1 Division of Orthodontics, Faculty of Medicine, Medical University of Gdańsk, Aleja Zwycięstwa 42c, 80-210 Gdańsk, Poland; 2 University Dental Center, Medical University of Gdańsk, Dębowa 1a Street, 80-204 Gdańsk, Poland

**Keywords:** CBCT, cone-beam computed tomography, temporary anchorage devices, miniscrew, infrazygomatic crest, IZC, maxilla, maxillary sinus, orthodontics, orthodontics anchorage procedures

## Abstract

There is no unequivocal scientific consensus for the temporary anchorage device (TAD) positioning in the infrazygomatic crest area (IZC). The two principal aims of this systematic review were to assess bone availability in the IZC and to establish both the target site and the need for cone-beam computed tomography (CBCT) prior to miniscrew placement. The study was performed following PRISMA guidelines (PROSPERO: CRD42023411650). The inclusion criteria were: at least 10 patients, three-dimensional radiological examination, and IZC assessment for the TAD placement. ROBINS-I tool and Newcastle-Ottawa Scale were used for quality evaluation. No funding was obtained. The study was based on the information coming from: PubMed, Google Scholar, Web of Science Core Collection, MDPI, Wiley, and Cochrane Libraries. The last search was carried out on 1 August 2023. Fourteen studies were identified for analysis. A narrative synthesis was performed to synthesize the findings of the different studies. Unfortunately, it is not possible to establish the generally recommended target site for IZC TAD placement. The reasons for this are the following: heterogeneity of available studies, inconsistent results, and significant risk of bias. The high variability of bone measurements and the lack of reliable predictors of bone availability justify the use of CBCT for TAD trajectory planning. There is a need for more high-quality studies aiming three-dimensional bone analysis of the IZC.

## 1. Introduction

Skeletal anchorage has expanded orthodontic treatment possibilities and is an integral part of modern orthodontic practice [1]. Temporary anchorage devices (TADs) can be placed within the maxilla in several locations such as the alveolar process [2,3], tuberosity [4], palate [5,6,7,8,9], and infrazygomatic crest area (IZC) [10,11]. Extra-alveolar miniscrews offer benefits such as reduced risk of root damage and the lack of interference with the mesiodistal tooth movement [11,12,13]. The IZC and external oblique ridge (the so-called buccal shelf) are the most frequently used extra-alveolar sites [11,14]. The TADs placed into the IZC region have been successfully used for total arch maxillary distalization with clockwise rotation of the occlusal plane [15]. Moreover, miniscrews in the IZC region seems to be more cost-effective than the palate distalizers, which offers a similar amount of tooth movement [13,15,16].

**Figure 1:**
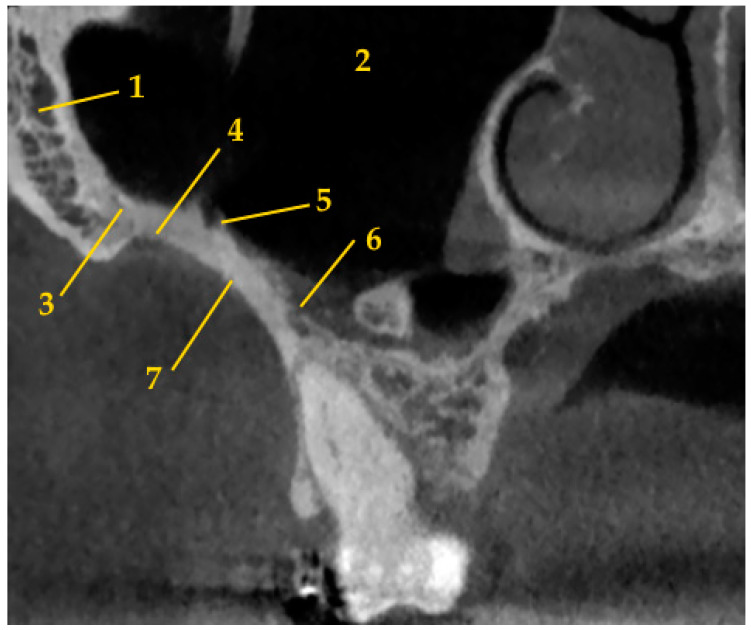
The anatomy of infrazygomatic crest area (1—zygomatic bone; 2—maxillary sinus; 3—zygomatico-maxillary suture; 4—zygomatic process of maxilla; 5—posterior superior alveolar artery; 6—posterior superior alveolar vein; 7—infrazygomatic crest).

The IZC is a cortical bony eminence which is located on both sides of the zygomatic bone to the alveolar process at the level of the first molar or between first molar and second premolar (Figure 1). The IZC TADs are usually placed according to the clinical protocol, which allows to place miniscrews in the upright position in the lateral part of the posterior alveolar process between the first and second maxillary molars and to omit slipping on the bone surface [11]. However, some authors preferred to place IZC TADs in the “key ridge” above the first permanent molar [10]. Moreover, miniscrews could be placed with multiple angle adjustment technique in order to obtain more vertical orientation [17].

The head of the miniscrew should be positioned at least 5 mm superior to the level of the soft tissue in order to facilitate oral hygiene and control soft tissue irritation [11]. It seems that the TAD should not only be placed in attached gingiva and mucogingival junction areas, but also in the zone of opportunity, which ranges to 2 mm of the mucosa apical to the mucogingival junction. It is possible to obtain greater amount of bone here with improved interradicular distance and healing response of the mucosa, which is similar to that for the attached gingiva [18,19,20].

On the other hand, there is a high likelihood of sinus perforation after miniscrew placement in the IZC region [21,22]. If the TAD penetrates more than 1 mm into the sinus, there is significantly higher incidence of sinus membrane thickening [21]. Concomitantly, the success rate of miniscrews placed into the IZC region is controversial and amounts from 78.2% to 96.7% [10,11,21,22].

Three-dimensional radiological examination provides detailed information about alveolar bone morphology [23]. Due to the lack of generally recommended target site and ambiguous success rate of IZC miniscrews, it was decided to perform this systematic review on papers evaluating IZC anatomy by means of three-dimensional radiological examination. The chosen research questions were as follows: What is the bone availability in the IZC area? What is the recommended target site? Should cone-beam computed tomography (CBCT) be performed prior to IZC orthodontic TAD placement for more predictable results?

## 2. Materials and Methods

Preferred Reporting Items for Systematic Reviews and Meta-Analyses (PRISMA) guidelines were used in this study [24]. The study protocol was registered with the Prospective Register of Systematic Reviews (PROSPERO; CRD42023411650). A narrative synthesis was performed to synthesize the findings of the different results of various studies.

### 2.1. Search Criteria

#### 2.1.1. Inclusion Criteria

As a template to formulate a clinical question the PICO (P—population, I—Intervention or exposure, C—Comparison, O—Outcome) was used. Study characteristics:

P. At least 10 patients
I. Three-dimensional radiological examination
C. Not required
O. Assessment of IZC for orthodontic miniscrews placement

Only those papers written in English and study only on human were enrolled for reviewing.

#### 2.1.2. Exclusion Criteria

Articles not meeting the PICO criteria were excluded from the systematic review. Publications that assessed the bone support after TAD placement were excluded due to two reasons—potential metal artifacts caused by the appliance and assessment driven by final placement instead of regional anatomy.

### 2.2. Data Collection

In order to find relevant studies, international databases including PubMed, Google Scholar, Web of Science Core Collection, MDPI, Wiley, and Cochrane Libraries were searched. We analyzed the scientific literature concerning IZC and three-dimensional radiological examination available from 2007 to the 1st of August 2023. The search terms used for the review were: [(“maxilla” OR “infrazygomatic crest”) AND (“mini-implant” OR “miniscrew” OR “TAD” OR “temporary anchorage device” OR “skeletal anchorage” OR “orthodontics anchorage procedures”) AND (“orthodontics” OR “Tooth Movement Techniques”) AND (“cone-beam CT” OR “cone beam computed tomography”)] and [(“infrazygomatic crest” OR “infrazygomatic crest area” OR “IZC”) AND (“mini-implant” OR “mini-implants” OR “miniscrew” OR “TAD” OR “temporary anchorage device” OR “skeletal anchorage” OR “orthodontics anchorage procedures”) AND (“cone-beam CT” OR “cone beam computed tomography”)] and [(“maxilla” OR “infrazygomatic crest”) AND (“mini-implant” OR “miniscrew” OR “TAD” OR “temporary anchorage device” OR “skeletal anchorage” OR “orthodontics anchorage procedures”) AND (“orthodontics” OR “Tooth Movement Techniques”) AND (“CT” OR “computed tomography”)] and [(“infrazygomatic crest” OR “infrazygomatic crest area” OR “IZC”) AND (“mini-implant” OR “mini-implants” OR “miniscrew” OR “TAD” OR “temporary anchorage device” OR “skeletal anchorage” OR “orthodontics anchorage procedures”) AND (“CT” OR “computed tomography”)]. References were imported into Mendeley manager. In order to identify eligible studies for the review, the papers were screened basing on titles and abstracts. Afterwards, full-text articles were assessed for eligibility.

Subsequently, data were extracted from those records retrieved for detailed text evaluation. These procedures were conducted by the first author. The second author participated in cases of disagreement. The following information was collected: first author, year of publication, study type, group characteristic (number, age, sex, ethnicity), aim of the study, assessment methods, bone measurements, and conclusions. Duplicate records, as well as letters and papers that did not contain significant information, were also excluded. Subgroup analyses were used to explore possible causes of heterogeneity among study results.

### 2.3. Quality Assessment

In this article the risk of bias was assessed of the included studies using the ROBINS-I tool (Risk of Bias in Non-randomized Studies of Interventions) and Newcastle-Ottawa Scale (NOS). ROBINS-I evaluates the following domains: (1) confounding; (2) selection of participants; (3) classification of interventions; (4) deviations from intended interventions; (5) missing data; (6) measurement of outcomes; and (7) selection of reported result (Table 1) [25]. Then each of the risk of bias domains was classified as: low, moderate, serious, critical, or no information. Overall risk was scored in the same gradation. NOS scale assess: (1) study selection; (2) comparability; (3) exposure (Good quality: 3 or 4 stars in sample selection category AND 1 or 2 stars in comparability category AND 2 or 3 stars in exposure category; Fair quality: 2 stars in sample selection category AND 1 or 2 stars in comparability category AND 2 or 3 stars in outcome; Poor quality: 0 or 1 star in sample selection category OR 0 stars in comparability category OR 0 or 1 stars in exposure category) [26]. These procedures were performed by both authors. In case of disagreement, a consensus reading was made.

## 3. Results

### 3.1. Literature Search

**Figure 2:**
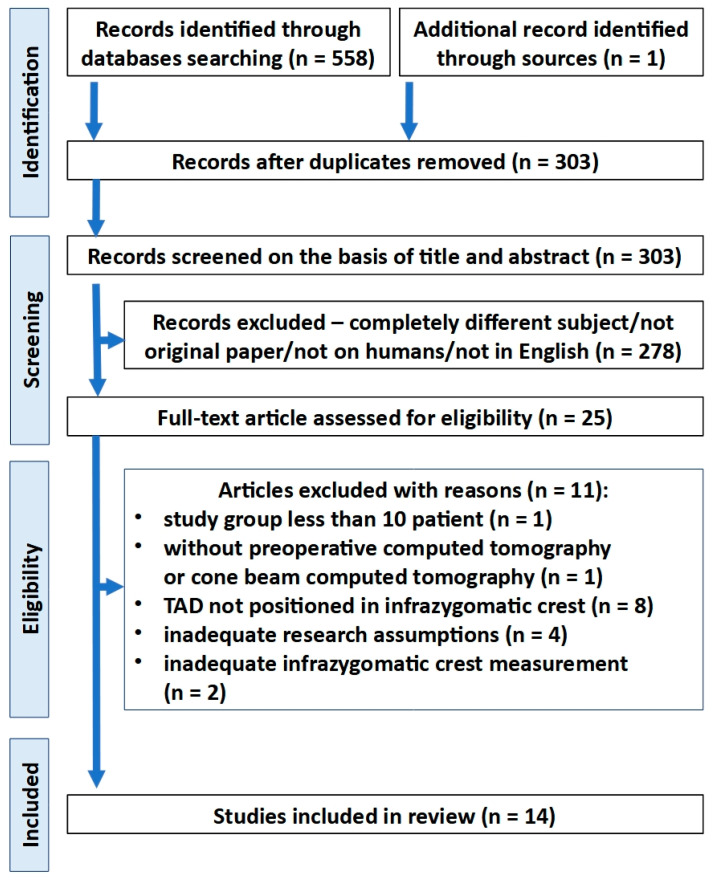
PRISMA flow diagram depicting the process followed for the selection of the studies.

In the first stage of selection, a total of 303 records were identified after duplicated references had been removed. Twenty-five studies were retrieved for full-text detailed evaluation. Next, 11 articles were excluded. Of these, two studies were excluded due to inappropriate assessment method, which did not take into consideration the recommended IZC TAD position [19,27]. Finally, 14 articles were included in the review (Figure 2 and Table 2).

### 3.2. Study Characteristic

All of the studies were retrospective and cross-sectional. There were five studies from Brazil, three from China, and single studies from Taiwan, USA, Saudi Arabia, India, Colombia, and Poland. The biggest study group consisted of 128 patients [36]. Only one and concomitantly the oldest paper [28] used spiral computed tomography (CT) for IZC anatomy evaluation. The rest of the studies (n = 13) performed the measurements by means of CBCT. Seven papers evaluated the IZC bone availability in relation to sex [29,31,32,35,36,38,40] and five investigated age differences [29,32,36,38,40]. Five studies analyzed the IZC anatomy in relation to different vertical skeletal patterns [12,33,34,37,39] and one in relation to different sagittal skeletal patterns [39]. One study evaluated the bone availability in relation to regional anatomy—modified palatal height index [40].

There were different measurement methods for IZC bone availability. Most popular assessment sites were mesiobuccal and distobuccal roots of maxillary first molar (n = 8), and region between maxillary first and second molars (n = 6). Other assessment sites were regions between maxillary second premolar and first molar, middle of the upper first molar, and mesiobuccal root of maxillary second molar (n = 3), distobuccal root of maxillary second molar (n = 2), and middle of the upper second molar (n = 1). Eleven studies evaluated the overall bone depth, three the bone thickness, and one the bone height in the IZC region. Single studies evaluated the buccal cortical bone depth and the buccal cortical bone thickness. Seven papers investigated different insertion regions. Eight studies investigated different insertion heights and seven compared different transversal angulations. Only one of the studies examined different sagittal angulations.

High variability of the results was found within individual studies as well as between different studies. Individual sample differences were high, as indicated the standard deviation which was often around 50% or more of the mean.

There was no consensus about best insertion region among studies, which evaluated the bone depth in different sagittal insertion sites [33,34,36,38,40]. Two studies recommended distobuccal root of first molar [36,38], one study mesiobuccal root of first molar [34], one mesiobuccal root of second molar [33], and one region between maxillary first and second molars [40]. Concomitantly, Arango et al. [36] recommended to place TADs in the region between second premolar and first molar in younger patients.

Moreover, there was no agreement among studies, which evaluated transversal angulations influence on the bone depth availability [28,32,34,35,36,38,39]. Only four studies performed statistical evaluation of the obtained results. On the one hand, Amri et al. [32] obtained significant negative correlation with the bone depth decrease when the insertion angle increase. On the other hand, Du et al. [35] found significant positive correlation in most sites. Instead, Tavares et al. [39] found no significant differences between different angulations at the same insertion level. Song et al. [38] assessed all of the possible angulations from 0 to 90° and obtained significant differences. The highest values were related to the transversal angulations from 60° to 70°. The transverse angulation was recommended to be larger at lower insertion heights and concomitantly the angle could be relatively smaller at higher insertion sites [28,38].

Paper, which assessed sagittal angulation effect on the bone depth availability, revealed that the bone depth increased with the higher sagittal inclination and this relation was not statistically significant only in one insertion path [35].

There was a tendency to bone depth decrease in the apical direction [12,35,38,39,40]. Concomitantly, in two studies which compared levels in proximity, not exceeding a distance of 2 mm between sites, no significant differences were found [12,39]. On the other hand, Gibas-Stanek et al. obtained significant differences in the region of distobuccal roots of maxillary first molar and region between maxillary first and second molars [40]. Du et al. [35] and Song et al. [38] also obtained statistical significance. The former compared the measurements performed in three levels within a distance of 4 mm and obtained significant negative correlation between insertion height and bone depth. The latter performed measurements at thirteen levels within distance of 12 mm. The results suggested an optimal range of insertion heights.

Both studies, which assessed bone thickness in various insertion regions, recommend to placing TADs more distally in the region between first and second maxillary molars or even in the region of mesiobuccal root of second maxillary molar [30,37]. One study investigated the relation between angulation and bone thickness [35]. The bone thickness increased significantly with higher transversal angulations. Concomitantly, no significant differences in bone thickness were found among different sagittal insertion angles.

There was a tendency to bone thickness increase in the apical direction [30,35,37]. Statistical significance was assessed and found in two studies [35,37]. Moreover, Lima et al. [37] found significant differences in the buccal alveolar bone thickness measurements, which tend to get higher from the region between upper premolars to the region between molars. In the same study were the alveolar bone heights evaluated. The lowest alveolar bone height was found between molars and the highest was found between premolars. However, no significant differences were obtained [30].

Only one of the studies evaluated both the bone depth and the bone thickness [35]. A statistically significant and negative correlation between these measurements was found in the study.

There was no agreement about recommended insertion height (Table 3) [12,28,30,31,35,37,38,39,40].

No statistically significant differences were found between different genders in most of the studies which assessed that relationship [29,31,32,35,36]. Song et al. [38] obtained statistically significant differences, but these differences were concomitantly clinically insignificant. Gibas-Stanek et al. [40] obtained statistically significant differences only in two insertion heights (14 mm and 16 mm above the occlusal plane) in the region between maxillary first and second molars. These differences, which were both 1.2 mm, also seem to be clinically significant.

There was no consensus about age differences in IZC bone depth among studies, which obtained contrary results [36,38,40]. Al Amri et al. [32] found no significant age-related differences in bone depth, but the study group consisted of only adult patients, which were 18 years or older. Moreover, there was no consensus about differences in cortical bone dimensions in the IZC area between the adolescents and the adults [29,38].

Moreover, no consensus on vertical growth pattern differences was found [12,33,34]. There were reports, that this relationship may be related to specific sites. Two of the studies did not statistically evaluate differences between the results obtained in different growth pattern groups [37,39].

A significant and negative correlation between bone depth and modified palatal height index was found only in 30% of the measurement sites [40].

Only one of the included studies performed not only static but also dynamic evaluation by taking into consideration distal movement trajectory of roots during orthodontic treatment [35].

CBCT imaging provides accurate clinical guidance for orthodontic TAD insertion. Six studies discussed the use of CBCT prior to IZC miniscrew placement [12,31,32,34,36,40]. There was an agreement among these studies that CBCT is justified for extra-alveolar TADs placement planning. The reasons were individual variation in growth and development of the maxilla and maxillary sinus, high variability of bone measurements, high risk of maxillary sinus perforation and the fact, that facial type is not a good predictor of bone availability.

### 3.3. Risk of Bias

#### 3.3.1. ROBINS-I

**Figure 3:**
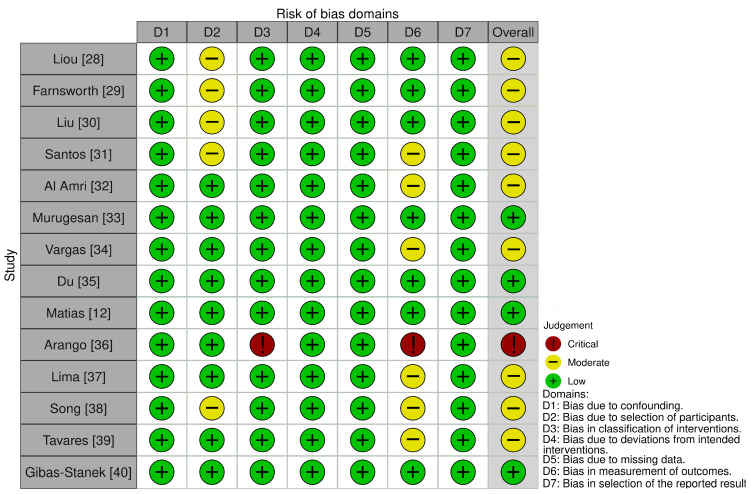
Risk of bias assessment using ROBINS-I tool [12,28,29,30,31,32,33,34,35,36,37,38,39,40].

Of the fourteen included studies, one was judged to be at critical risk of bias, nine were rated as having a moderate risk of bias and four had a low risk of bias. Main concerns related to the risk of bias were due to no reliable analysis in the intra-examiner assessment and no prospective calculation of the study size. In the study of Song et al. [38], should have the eligible patients had the distance between maxillary sinus and alveolar ridge crest no less than 10 mm. It is a potential reason of incredible results due to overestimation. In the study of Arango et al. [36] was one of the measurements, IZC region bone length performed in three sagittal sites and in three different angulations, not described. Concomitantly, the study of Liu et al. [30] which was referenced in the above publication, did not present such a measurement. Therefore, that measurement was omitted in Table 2. The results of risk of bias assessment are shown in Figure 3.

#### 3.3.2. Newcastle-Ottawa Scale (NOS)

It was considered that one study is of good quality and 13 are of fair quality. The risk of bias assessment using the NOS was described in Table 4.

## 4. Discussion

Recent development in three-dimensional X-ray diagnostics have enabled more precise measurements of alveolar bone structure [41]. Concomitantly, CBCT provides lower radiation dose than spiral CT [42]. Only four studies included in the review discussed the radiation dose during the CBCT examination [32,34,36,39]. Current guidelines state, that CBCT is not normally indicated for planning the placement of TADs in orthodontics. This examination is needed preoperatively for placement of miniscrews in cases of borderline dimensions [43]. High variability of the results was associated with different root lengths, anatomy of the maxilla and the maxillary sinus, transversal inclination of the adjacent teeth, and height of the alveolar process [30,31,33,35,37,38,39,40]. However, not only high variability of IZC bone architecture, but also no reliable predictors of bone availability were found during the review. It seems that only CBCT with adequate parameters can provide information about the bone amount, possibility of IZC miniscrew placement and preferred insertion path. It seems that insertion of the IZC TAD needs to be patient specific and one site is not adequate for all. Therefore, CBCT may be necessary to install IZC miniscrews correctly. It enables one to develop individual TAD insertion protocol for each patient to obtain desirable primary stabilization for orthodontic anchorage and to reduce complication risk. Not only insertion height and depth but also bone thickness, cortical bone amount and different transversal and sagittal angulations should be taken into consideration. When considering favorable risk-benefit ratio, it seems justified to use CBCT for IZC miniscrew placement planning. However, radiological protection is needed for this type of examination especially in young patients [44]. It is recommended to reduce the field of view to the maxilla region if there are no other indications for increasing the imaging area size. This approach is in conformity with the ALARA (as low as reasonably achievable) principle. This rule involves maintaining exposures to radiation as far below the dose limits as is practical, while being consistent for which the activity is undertaken [45].

According to the literature, decreasing the CBCT voxel size can improve the accuracy of alveolar bone measurements [44]. Examination with a 0.2-mm voxel size provides on average spatial resolution of 0.4 mm. Therefore, it can distinguish objects with a minimum 0.4-mm distance [46]. It provides clearer images, easier identification of alveolar crests, and enables closer to the gold standard (direct measurements) results [47]. Voxel size of approximately 0.4 mm can potentially be a limitation due to insufficiency accurate measurements [40]. On the other side, Farnsworth et al. [29] showed high reliability for both the 0.2 mm and 0.4 mm scans with high interclass correlations.

The positioning of the orthodontic miniscrew in the IZC area relative to the anatomical structures of the maxilla is of great importance. Only with the use of CBCT, it is possible to precisely determining the availability of the bone limited by the maxillary sinus floor location or the position of the posterior superior alveolar artery and vein. Moreover, the presence of septa in the maxillary sinus can be used as an additional bone support for the IZC TAD stability. The prevalence of sinus septa is estimated at 31.7% [48]. The posterior superior alveolar artery is located on or in the lateral wall of the maxillary sinus. The intraosseous variant is usually located at a height of 19 mm from the bone crest, and the extraosseous variant at a height of 23 mm from the bone crest. The average distance from the bottom of the maxillary sinus is about 6–9 mm. Moreover, the position of posterior superior alveolar vein should be also taken into consideration. It is in a more inferior position, which is more adjacent to the IZC insertion site than the artery. Both vessels can be damaged during the positioning of the miniscrew. This can lead to postoperative complications in the form of prolonged bleeding [49,50,51]. Jia et al. [21] assessed the incidence of TADs penetration into the maxillary sinus and penetration depth influence on sinus tissue. The frequency of penetration of miniscrews into the maxillary sinus was high. The incidence of membrane thickening was 88.2% in a group in which penetration exceeded 1 mm, and the mean value of thickening was 1 mm. In a group with membrane penetration of 1 mm was the membrane thickening observed at 37.5%, and the mean value of thickening was 0.2 mm. According to Chang et al. [22] perforation of the sinus reduced both the length of the bone contact and the terminal insertion torque, but did not significantly increase the failure rate of TADs.

Thick cortical bone is associated with good primary stability. Therefore, it is not recommended to place the miniscrews in thin cortical bone, which is less than 1 mm thick [52]. However, there is no consensus how does the insertion torque affect the survival rate of orthodontic TADs [22,52]. Placement failure might be associated with low as well as excessive torque values [52]. IZC are might be expected to undergo greater damage, such as crushing and heat, due to thick cortical bone during placement [29]. Pilot holes might be preferred to omit this potential damage in certain patients [29,52].

Nine studies presented requirements for adequate bone availability [28,33,34,35,36,37,38,39,40]. There was no consensus about preferred values of the bone measurements. Liou et al. [28], Arango et al. [36], Tavares et al. [39], and Gibas-Stanek et al. [40] suggested the required bone depth value of 6 mm. Concomitantly, all of the patients met this requirement in the study of Liou et al. Moreover, mean bone depths obtained the required value except one insertion path in class 2 patients in the study performed by Tavares et al. [39]. However, significant variability was noticed. Whereas Arango et al. obtained a significant bone variability with mean measurements beneath the adopted limit value. Gibas-Stanek et al. found that bone depth rarely reaches recommended value of 6 mm. Murugesan et al. [33] and Song et al. [38] required bone depth of 5 mm. In the former study was that mean bone amount not obtained only in high angle patients’ group above the mesiobuccal root of first molar. The mean bone depth was adequate above the mesiobuccal root of a second molar in all skeletal patterns. In the latter study was the recommended areas, with greater bone depth than required 5 mm, presented. There is a possibility, that border value of 5–6 mm might be exaggerated and significantly limits the choice of sites for miniscrew safe insertion in the IZC region. Therefore, Vargas et al. [34] and Du et al. [35] preferred values of 4 mm and 3.8 mm, respectively. Most of the measurements in the IZC were lower than 4 mm in the former study. In the study of Du et al., the median bone depths of most paths did not reach 3.8 mm only at the 17 mm insertion height. Farnsworth et al. [29], following the study of Motoyoshi et al. [52], preferred the cortical bone depth of 1 mm. Most of the patients met this condition [29,38]. Other requirements were presented according to the bone thickness measurements. Lima et al. [37] preferred bone thickness of 3 mm, when Du et al. [35] recommended 1.3 mm. This value was calculated as a sum of 0.8 mm TAD radius and 0.5 mm safety distance from root surface. According to Lima et al. mean values of bone thickness were larger than 3 mm between first and second molar, and in the mesial second molar root area 11 mm from the alveolar crest. In the study of Du et al. [29], was the median bone thickness of each path at 13 mm height less than 1.3 mm when transversal angulation of 50° was adopted. A statistically significant and negative correlation between the bone depth and the bone thickness was found. Therefore, both measurements should be taken into consideration for insertion path analysis. The extent of the results and obtained standard deviations in sites with adequate mean bone values indicate that there is no universality of the measurements and some patients presented inadequate bone availability. There is a need for comprehensive studies considering different aspects of regional bone anatomy to establish the bone requirements. The aspects of bone quantity and quality with possible bicortical fixation that could increase the clinical success should be considered.

Dynamic evaluation presented by Du et al. [35] seems to be more adequate for orthodontic purposes than static measurements. Potential collision of the miniscrew with orthodontic tooth movement may prevent the clinician from obtaining the desired treatment results and may lead to root injuries or failure of the miniscrews. Therefore, continuous evaluation taking into consideration distal movement trajectory of roots is justified. Future studies should take this approach into consideration.

Orthodontic biomechanics should be also considered when IZC miniscrew placement planning. Tooth movement patterns during total maxillary arch distalization with different force directions were recognized [53]. None of the included studies discussed in this aspect of miniscrew placement. More distal and occlusal placement sites provide higher relation between values of horizontal and vertical force vectors when comparing with mesial and more apical placement sites. Therefore, power arms should be more frequently considered in forward and apical locations.

There was no consensus that higher transversal angulations were associated with greater bone depth. On the other hand, it seems that it is better to implant TADs closer to the occlusal plane to ensure the appropriate depth of implant insertion, and the apically increasing bone thickness provides support and distance to root surfaces despite transverse angulation of the miniscrew. This also seems to be advantageous considering the biomechanical aspects. Moreover, additional stabilization increase may be obtained due to miniscrews’ sagittal angulation. However, more acute insertion angle is associated with more technically difficult placement due to slipping [28]. Precise TAD insertion may still be a challenge for manual manipulation, especially for inexperienced physicians. Du et al. [35] indicated a need for assistive device or intelligent robot to be developed. Implementation of optical scanning and 3D printing to fabricate customized appliances including guides for patients with craniofacial disorders was proved to be efficient way in the individualization of the treatment [54]. Moreover, there is a possibility to use insertion guides for more predictable results [6,55]. Surgical guides for IZC miniscrew placement can be obtained based on CBCT examinations and intraoral scans. Implementation of optical scanning and 3D printing to fabricate customized appliances including guides for patients with craniofacial disorders is proved to be efficient way in the individualization of the treatment. This approach could potentially improve the primary stability and reduce both the TAD collision risk during orthodontic tooth movement and the sinus penetration risk. Moreover, it could solve the slipping problem during insertion. The use of surgical guides for IZC miniscrew placement should be further researched.

An interesting issue is the use of artificial intelligence-supported determination of available sites for orthodontic miniscrews based on bone analysis using the CBCT [56,57]. Today, it is not common in dentistry and orthodontics. The AI system demonstrated high accuracy in bone segmentation and measurement, which is helpful in identifying available sites and designing a surgical template for palatal TADs [56]. The use of AI for IZC miniscrew placement planning should be researched in the future.

The differences of IZC TADs’ success rates among previously mentioned studies might be caused by different localizations and miniscrews’ dimensions. Uribe et al. [10] preferred the “key ridge” above the first permanent molar, where reduced bone depth due to high insertion position could be present. These authors favored also TADs of smaller width than the other authors did [11,21,22]. Both factors seem to affect the obtained results. There is no consensus on the ideal IZC position in terms of vertical dimension. Some authors propose positioning relative to the occlusal plane [12,35,40,58]. It may not be a reproducible place for positioning TADs in cases of pathological tooth wear or disturbed occlusal plane. Also, the height of the crown of the teeth varies. The use of the mean height of clinical crowns measured between the cusp tip and the cemento-enamel junction is not reliable due to individual variability. Another aspect is the clinical significance of the variables, which potentially could be insignificant in case of minor deviation. Moreover, adjustment of the single molar occlusal plane as the referential [33,39] seems to be even more limited due to the common compensatory transversal inclination of the molars. A repetitive location could be positioning using the mucogingival line as a landmark. As a rule, miniscrews should be located within the safe zone adjacent to the mucogingival line. This protects the oral mucosa from excessive exposure to inflammation caused by the implant irritating the mobile mucosa. Due to both diverse gingival biotypes of patients and different height of keratinized gingiva, it is difficult to define uniform standards for the positioning of TADs. Alveolar crest in the condition of healthy periodontium or cemento-enamel junction should be considered as the potentially good referential site. Establishing a reference value would allow more accurate positioning of miniscrews.

Cross-sectional studies have limited reliability in the age-related assessments. Therefore, it is justified to perform cohort studies. However, there are ethic aspects as well as radiological protection requirements, which should be taken into consideration when performing two CBCT examinations in the untreated study group.

Only in the study of Santos et al. [31] was a need for maxillary anchorage during orthodontic treatment stated as an inclusion criterion.

The available literature lacks a systematic review on the positioning TADs in the IZC area. The presented study shows limitations of existing evidence, new directions of research, and their degree of advancement on the way to implementation in everyday practice. The limitation of this article is the scarcity of literature reports on this subject. The most studies included in the systematic review was the assessment of the location of miniscrews in IZC without clear standards. The studies differ in the methods of measuring the depth and angulation of the inserted TADs, the level of insertion relative to the horizontal plane, the diversity of patients in terms of ethnic group or age of patients. Moreover, the IZC position was commonly not assessed in the absence of maxillary molars or the presence of significant congenital disorders such as cleft palate. The influence of the alveolar bone loss due to periodontal disease on the bone depth in IZC region was not investigated. Also, periodontal biotype and recessions presence were not evaluated as potential prognostic factors of the bone availability. These aspects should be researched in the future.

## 5. Conclusions

It is not possible to establish the generally recommended target site for the placement of the miniscrews in the IZC area. The reasons for this were the heterogeneity of available studies, inconsistent results, and significant risk of bias. The high variability of bone measurements and the lack of reliable predictors of bone availability justify the use of CBCT for TAD trajectory planning. Finally, there is a need for more high-quality studies aiming three-dimensional bone analysis of IZC area.

## Figures and Tables

**Table 1:** Criteria adopted for risk of bias assessment using ROBINS-I tool.

Domains	Criteria
**Bias due to confounding**	Studies are considered at **low risk** of bias if they consider the bone measurements at common sites of TAD placement in the IZC. Studies are considered at **moderate risk** of bias if they consider the presence of maxillary deciduous molars or the absence of maxillary permanent molars as confounding factors. Studies are considered at a **high risk** of bias if adjustment factors are not reported.
**Bias in selection of participants into the study**	Studies are considered at **low risk** of bias if the study size population was performed prospectively. Studies are considered at **moderate risk** of bias if the exposed cohort has some representation of the assessed exposure. Studies are considered at **high risk** of bias if the exposed cohort does not faithfully represent the assessed exposure.
**Bias in measurement of the interventions**	Studies are at **low risk** of bias if a correct measure of IZC bone is reported on CBCT or CT scans. Studies are considered at **moderate risk** of bias if the measurement of IZC bone is not performed on CBCT or CT scans. Studies are at **high risk** of bias if the way of measuring of IZC bone was not reported.
**Bias due to departures from intended interventions**	Studies are at **low risk** of bias if no intervention in the maxillary arch was performed, while at **moderate risk** of bias if any intervention in the opposite arch to change the reference point was performed. Studies are considered **high risk** if any intervention was performed on the maxillary arch (leveling, arch expansion, sagittal tooth movement).
**Bias due to missing data**	Studies are considered at **low risk** of bias if less than 10% of participants were excluded to missing data, while at **moderate risk** of bias if less than 20%. Studies with higher proportion (≥20%) are considered at **high risk** of bias.
**Bias in measurement of outcome**	Studies are at **low risk** of bias if raters are aware of the limitations of the study or method error has been statistically evaluated. Studies are considered at **moderate risk** of bias if no reliability analysis has been performed in the intra-examiner’s assessment. Studies are at **high risk** of bias if the outcome assessment is based solely on self-report, without external validation.
**Bias in selection of reported results**	Studies are at **low** risk of bias if all data planned by the authors in the entire sample are analyzed in accordance with a prescribed plan. Studies are considered at **moderate** risk of bias if there is no information about a prespecified plan. Studies are at **high** risk of bias if numerical result being assessed likely to have been selected on the basis of the results.
**Overall risk of** **bias**	If at least one domain was found at **high** risk of bias, the overall risk was considered **high**. If at least one domain is at some concerns, but no domains are at high risk, the overall risk was considered **moderate**. If all domains were at **low** risk of bias, the overall risk was considered **low**.

**Table 2:** The studies included in the qualitative analysis and extracted data.

No	Study	Year	Study Type	Patient Characteristic			Aim	Assessment Method			Main Results—IZC Bone Measurement	Statistical Significance	Conclusions
				Country	Number	Age		Radiographical Examination	Insertion Region	Measurement Type and Method			
1	Liou [28]	2007	CSS	Taiwan	16 (M: 10, F: 6)	27.0 ± 5.2 y	The IZC depth of above the first maxillary molars, the biting depth of IZC TAD at different angles and positions to the maxillary OP, clinical implications for TAD insertion in the IZC without injuring the 6 MB root	Spiral CT	6M	Depth, eight transverse angulations: 40–70° (5° increments) to the maxillary OP, apical measurement point was the sinus point (intersection of tangent to the tooth buccal surface reference line and the floor of the maxillary sinus), 17.1 ± 3.7–12.8 ± 4.2 mm above the OP	40°: 5.2 ± 1.1 mm 45°: 5.4 ± 1.1 mm 50°: 5.6 ± 1.2 mm 55°: 6.0 ± 1.4 mm 60°: 6.3 ± 1.5 mm 65°: 7.0 ± 1.7 mm 70°: 7.7 ±1.9 mm 75°: 8.8 ± 2.3 mm	No significant differences *(p* > 0.05) between different sides corresponding measurements	Ideal site (the adopted minimum depth of 6 mm), at an angle of 55–70° and 14–16 mm above the maxillary OP
2	Farnsworth [29]	2011	CSS	USA	52 (M: 26, F: 26)	Adolescents: 13 M: 14–16 y; 13 F: 11–13 y; Adults: 13 M; 13 F; 20–45 M and F	To examine the cortical bone thickness at common sites of TAD placement	CBCT, voxel size 0.39 mm	6M	Cortical bone thickness, one level: parallel to 6 M and 2 mm coronal to the junction of maxillary sinus and IZC cortical plates	Adolescents: 1.44 ± 0.39 mm; Adults: 1.58 ± 0.34 mm	No gender significant differences. No age significant differences	Cortical bones at commonly used TAD placement sites are thicker in adults than in adolescents except IZC
3	Liu [30]	2017	CSS	China	60 (M: 18; F: 52)	26.0 ± 7.8 y	To study the thickness and height of the alveolar bone in the IZC via the CBCT technique and to provide guidelines for choosing the appropriate TAD and the safe zone for TAD placement	CBCT, 110 kV, 0.07 mA, voxel size 0.3 mm	5–6, 6IR, 6–7	Thickness and height, five levels: 4 horizontal (5, 7, 9, 11 mm above the alveolar crest, parallel to OP), 1 vertical (parallel to the long axis of maxillary first molar)	Thickness: 5–6: 1.56 ± 0.24–1.86 ± 0.36 mm, 6IR: 2.24 ± 0.58–3.05 ± 0.58 mm, 6–7: 3.04 ± 0.55–4.07 ± 0.74 mm. Height: 5–6: 12.41 ± 5.59 mm, 6IR: 10.63 ± 4.4 mm, 6–7: 10.36 ± 3.38 mm	Significant difference in the thickness among the regions at the same plane (*p* < 0.05), no significant difference for thickness at different planes of each region (*p* > 0.05), no significant difference in height among the regions (*p* > 0.05)	6–7 should be the first choice for TAD placement in the buccal alveolar bone in the IZC for distalization of the entire maxillary dentition. Proper length of TAD is 6–8 mm for most patients. Bone tendency to get thicker apically
4	Santos [31]	2017	CSS	Brazil	40 (M:18; F:22)	22–56 y (mean 31 y)	To evaluate IZC depth in adult patients	CBCT, 120 kVp, 47 mA, voxel size 0.4 mm, FOV 20 × 25 cm	6D	Depth, two levels: 2 mm and 4 mm above FP, angulation 90 ° to the cortical surface	2 mm: 2.49 mm 4 mm: 2.29 mm	No gender and side significant differences. Significantly higher depth at 2 mm level (*p* = 0.019). 2 mm no significant side differences (*p* = 0.111) and 4 mm significant side differences (*p* = 0.002)	IZC crest is significantly thinner than the length of the miniscrews. Risk of instability and sinus perforation due to insufficient bone depth
5	Al Amri [32]	2020	CSS	Saudi Arabia	100 (M:50, F:50)	25.4 ± 6.5 y	To assess the proximity of the maxillary sinus and nasal cavity in areas where miniscrews are usually inserted	CBCT, 120 kVp, 5 mA, voxel size 0.4 mm	6M	Depth, three transverse angulations: 45°, 55°, and 70° to the molar OP, apical measurement point was the sinus point (intersection of tangent to the tooth buccal surface reference line and the floor of the maxillary sinus)	45°: 4.94 ± 0.73 mm, 55°: 3.73 ± 0.41 mm, 70°: 3.90 ± 0.31 mm	No gender and age significant differences. IZC crest bone was significantly thicker at an insertion angle of 45° than at 55° and 70° (*p* < 0.001)	Greatest bone depth was at a 45° insertion angle. Risk of maxillary sinus perforation due to the limited available bone
6	Murugesan [33]	2020	CSS	India	36	20–30 y; average angle: 22.25 ± 4.31 y, low angle: 21.42 ± 4.52 y, high angle 21.5 ± 2.71 y	To compare the IZC crest depth in different vertical skeletal patterns (Jarabak index: S-Go and N-Me ratio and Tweed’s FMA)	CBCT	6M, 7M	Depth, 70° to the molar OP, apical measurement point was the sinus point (intersection of tangent to the tooth buccal surface reference line and the floor of the maxillary sinus)	Average angle: 6M: 6.66 ± 2.80 mm, 7M: 9.20 ± 2.23 mm; Low angle: 6M: 6.09 ± 1.82 mm, 7M: 7.88 ± 1.84 mm; High angle: 6M: 3.85 ± 0.65 mm, 7M: 6.66 ± 1.63 mm	No side differences. Bone depth significant differences between insertion regions. High angle and average angle (6M,7M), high angle and low angle (6M) significant differences (*p* < 0.001, *p* = 0.001, *p* = 0.001)	IZC bone depth varied among different vertical skeletal patterns. It was least thick in high-angle group. Recommended site is 7M
7	Vargas [34]	2020	CSS	Brazil	100 (M: 42; F: 58)	19.8 ± 5.5 y	To use CBCT to determine the bone thickness in the MBS and bone depth in the IZC in individuals with different vertical skeletal patterns (gonial angle: ramus line-mandibular line through the gnathion) for the ultimate placement of TAD	CBCT, voxel size 0.3 mm, FOV 18 × 20.6 cm	6M, 6D, 6–7	Depth, two transverse angulations: 65° and 70° to the OP, height localization at the apex level on the reference line tangent to buccal cortical bone	6M 65°: 3.5 (1.3–11.8) mm, 6M 70°: 3.6 (1.3–14.1) mm, 6D 65°: 2.8 (1.0–8.3) mm, 6D 70°: 3.0 (1.1–8.7) mm, 6–7 65°: 2.4 (1.0–7.0) mm, 6–7 70°: 2.5 (1.0–7.2) mm	Significant correlation between IZC bone depth with the gonial angle: 6–7 65° *p* = 0.013, 6–7 70° *p* < 0.001, no significant correlation in other sites (*p* > 0.05)	There was no correlation between the gonial angle and bone depth in the IZC. The best site to install IZC TAD is 6M
8	Du [35]	2021	CSS	China	35 (M: 16; F: 20)	23.1 y (20–28 y)	To measure the bone depth and thickness of different insertion paths for safe placement of IZC TADs between the 6–7 by 3-dimensional reconstruction and to explore their clinical significance	CBCT, 5 mA, 120 kV, FOV 14.0 × 8.5 cm, voxel size 0.2 mm	6–7	Depth and thickness, three levels: 13 mm, 15 mm and 17 mm above posterior OP, three transverse angulations (50°, 60°, 70°), three sagittal angulations (0°, 15°, 30°)	Depth: 13 mm50°: 8.00 ± 4,64 mm, 13 mm60°: 7.16 ± 2.61 mm, 13 mm70°: 7.33 ± 2.44 mm, 15 mm50°: 5.72 ± 2.92 mm, 15 mm60°: 5.52 ± 2.38 mm, 15 mm70°: 5.73 ± 2.28 mm, 17 mm50°: 4.04 ± 2.32 mm, 17 mm60°: 4.02 ± 2.12 mm, 17 mm70°: 4.24 ± 2.08 mm, 0° distal tipping angle	No significant differences between different sides and gender (*p* > 0.05). Significant differences in bone depth between different insertion heights (*p* < 0.001). Significant differences in bone thickness between different insertion heights and transverse angulations (*p* < 0.001). Significant negative correlation between IZC bone depth and bone thickness (*p* < 0.001)	Bone depth and bone thickness may affect the safe insertion of a TAD. TADs preferred insertion path: 15 mm above the posterior OP, transversal angulation of 60–70°, and sagittal angulation of 30°. Alternative path: 13 mm above posterior OP, transversal angulation of 70°, sagittal angulation of 30°. TAD should not be inserted 17 mm above posterior OP, or with transversal angulation of 50°. A TAD with 9–11 mm in length and 1.6–2.3 mm in diameter may be proper in IZC region
9	Matias [12]	2021	CSS	Brazil	45 (M: 20; F: 25)	22.2 ± 7.4 y (brachyfacial) 19.24 ± 5.92 y (mesofacial) 17.79 ± 3.63 y (dolichofacial)	To identify optimal areas for the insertion of TADs into the IZC and MBS, using CBCT imaging in patients with different vertical skeletal patterns (Ricketts VERT)	CBCT, 120 kV, 8 mA, FOV 13 × 16 cm, voxel size 0.30 mm	6D	Depth, three levels: 11 mm, 13 mm and 15 mm above the cusp tip; 70° to the maxillary OP	Brachyfacial: 11 mm: 9.33 ± 2.27 mm, 13 mm: 7.51 ± 2.16 mm, 15 mm: 5.94 ± 2.15 mm, Mesofacial: 11 mm: 8.82 ± 1.83 mm, 13 mm: 7.16 ± 1.93 mm, 15 mm: 5.59 ± 1.88 mm, Dolichofacial: 11 mm: 8.87 ± 1.91 mm, 13 mm: 7.11 ± 1.95 mm, 15 mm: 5.39 ± 1.86 mm	No side significant differences. There was no statistically significant difference in the IZC bone depth among the groups (*p* > 0.05)	The IZC bone depth was similar among the brachyfacial, mesofacial and dolichofacial groups. Bone decreased as the insertion height increased
10	Arango [36]	2022	CSS	Colombia	128 (M: 59; F: 69)	9–50 y (9–13 y, 14–23 y, 24–50 y)	To compare the dimensions of the ZP, IZC, and MBS by sex and age	CBCT,120 kV, 5 mA, voxel size 0.3 mm	5–6, 6IR, 6D	Depth, three transverse angulations: 55°, 65°, and 70° to the OP, apical measurement point was the sinus point (intersection of tangent to the tooth buccal surface reference line and the floor of the maxillary sinus)	IZC bone thickness: 5–6 at 55°: 3.06 (0.31–7.95) mm, 5–6 at 65°: 3.31 (0.31–8.84) mm, 5–6 at 70°: 3.53 (0.31–11.22) mm, 6IR at 55°: 2.62 (0.65–8.43) mm, 6IR at 65°: 3.16 (0.68–9.7) mm, 6IR at 70°: 3.78 (0.84–11.7) mm, 6D at 55°: 2.95 (0.48–8.95) mm, 6D at 65°: 3.35 (0.51–10.61) mm, 6D at 70°: 4.14 (0.51–11.4) mm	No gender significant differences. Significant differences were observed among age groups for IZC bone depth in 5–6 and 6IR (*p* < 0.001). No significant differences in 6D	IZC bone depth is bigger in younger subjects than in adults. 5–6 is the recommended site in the younger group and 6D in the older group
11	Lima [37]	2022	CSS	Brazil	86 (both sexes)	18–40 y	To evaluate the differences in bone thicknesses in the IZC among patients with different vertical skeletal patterns (Jarabak index: S-Go and N-Me ratio) to determine a safe zone for TAD insertion	CBCT, voxel size 0.4 mm	5–6, 6M, 6D, 6–7, 7M, 7D	Thickness; four levels: 5 mm, 7 mm, 9 mm, and 11 mm apically from a line passing through the mesial and distal alveolar bone crest	The required 3 mm minimum thickness: (1) hyperdivergent patients: 6–7 11 mm, 7M 9 mm and 11 mm, and 7D 11 mm; (2) neutral and hypodivergent patients: 6–7 11 mm and 7M 11 mm	All of the thicknesses measured at 5, 7, 9, and 11 mm presented statistically different means (*p* < 0.05) except left 5–6 in the hyperdivergent group	Safe zones for TADs—11 mm from the maxillary alveolar crest between the 6–7 and 7M for all of the facial types, thickness increase in distal and apical direction
12	Song [38]	2022	CSS	China	32 (M:16, F:16)	Adolescents: 14.4 ± 1.4 y Adults: 25.1 ± 5.5 y	To determine the optimal areas for IZC miniscrews	CBCT, 85 kV, 5 mA	6M, 6IR, 6D, 6–7, 7M, 7IR, 7D	Depth and cortical bone depth; thirteen levels: 0–12 mm (1 mm increments) from alveolar bone crest, ten transverse angulations: 0–90° (10° increments) to the OP	The most frequent shapes of IZC were the external concave shape, vertical shape, and external diagonal shape. Highest depth: 6D: 5.7 ± 5.2, 7D: 5.4 ± 4.7, Lowest depth: 6M: 3.8 ± 4.3	Cortical bone depth was significantly higher among females (*p* < 0.001). Depth was significantly higher among males (*p* = 0.01). Measurements were significantly higher among adults than adolescents (*p* < 0.001). Measurements were significantly influenced by insertion sites, heights, and angles	6D is the most ideal site. The optimal insertion heights and angles were 12 mm to 18 mm from the OP (3–9 mm from alveolar crest), and 40–70° for IZC miniscrews. Recommended insertion angle should be larger at lower insertion heights
13	Tavares [39]	2022	CSS	Brazil	58 (M:23, F:35)	29.5 ± 9.85 y (18–62 y)	To evaluate bone availability in the IZC crest in subjects with different vertical (SN-GoGn Angle) and sagittal (ANB) skeletal patterns	CBCT, 120 kV, 200 mA, FOV 32 × 32 cm, voxel size 0.6 mm	6D	Depth; three levels: 4 mm, 5 mm, and 6 mm from CEJ; three transverse angulations: 60°, 70°, and 80° to the molar OP	Bone depth was greater near the CEJ (8.7 ± 3.1 mm) and lower in the apical area (5.8 ± 2.7 mm)	Class 2—bone depth was significantly lower (*p* = 0.007) at 6 mm from CEJ at 80° when compared to 60° at 4 mm; Mesofacial subjects—bone depth was significantly lower at 80° at 6 mm from CEJ when compared to 60° at 4 mm	The best bone availability at the distance 4 mm from CEJ at an insertion angle of 60° for all individuals
14	Gibas-Stanek [40]	2023	CSS	Poland	100 (M:50, F:50)	28.81 ± 12.86 y (12–65 y)	To localize the most favorable site for IZC miniscrew implantation. To assess the dependency between bone availability, sex, and age	CBCT, 5 mA, 120 kV, FOV 13.0 × 15.0 cm, voxel size 0.38 mm	6M, 6D, 6–7 (left side)	Depth, three levels: 12 mm, 14 mm and 16 mm above OP; 70° to the maxillary OP	6M 12 mm: 2.5 ± 2.55 mm 6M 14 mm: 2.54 ± 2.42 mm 6M 16 mm: 2.42 ± 2.16 mm 6D 12 mm: 3.71 ± 2.76 mm 6D 14 mm: 3.11 ± 2.35 mm 6D 16 mm: 2.59 ± 2.08 mm 6–7 12 mm: 6.03 ± 2.64 mm 6–7 14 mm: 4.74 ± 2.17 mm 6–7 16 mm: 3.46 ± 1.93 mm	Site significant differences 12 mm and 14 mm above OP (*p* < 0.001). 6D and 6–7 height significant differences (*p* < 0.001). 6–7 14 mm and 16 mm gender significant differences (*p* = 0.012 and *p* = 0.003). 6M and 6D significant negative correlation with age (*p* < 0.001).	6–7 12 mm above the OP is the most ideal site. 6M 16 mm above the OP presented the lowest bone depth

CBCT—cone-beam computed tomography; CEJ—cemento-enamel junction; CSS—cross-sectional study; CT—computed tomography; F—female; FOV—field of view; FP—Frankfurt plane; IZC—infrazygomatic crest; M—male; MBS—mandibular buccal shelf; OP—occlusal plane; TAD—temporary anchorage device = miniscrew; ZP—zygomatic process; 5–6—region between maxillary second premolar and first molar; 6D—distobuccal root of maxillary first molar; 6IR—middle of the maxillary first molar; 6M—mesiobuccal root of maxillary first molar; 6–7—region between maxillary first and second molars; 7D—distobuccal root of maxillary second molar; 7IR—middle of the buccal furcation of the maxillary second molar; 7M—mesiobuccal root of maxillary second molar.

**Table 3:** Insertion height according to various authors.

No	Study	Recommended Insertion Height
1	Liou [28]	14–16 mm above OP
2	Santos [31]	2 mm above FP
3	Liu [30]	11 mm above AC
4	Du [35]	13–15 mm above OP
5	Matias [12]	11 mm above OP
6	Lima [37]	11 mm above AC
7	Song [38]	3–9 mm above AC
8	Tavares [39]	4 mm above CEJ
9	Gibas-Stanek [40]	12 mm above OP

AC—alveolar crest; CEJ—cemento-enamel junction; FP—Frankfurt plane; OP—occlusal plane.

**Table 4:** Risk of bias using the Newcastle-Ottawa Scale for quality assessment.

No	Study	Sample Selection				Comparability		Exposure		Total
		Adequate Case Definition	Representativeness of the Cases	Selection of Control	Definition of Control	Comparability of Cases	Controls Based on the Analysis	Ascertainment of Exposure	Non-Response Rate	
1	Liou [28]	**★**	**-**	**-**	**-**	**★**	**-**	**★**	**★**	4
2	Farnsworth [29]	**★**	**-**	**-**	**-**	**★**	**-**	**★**	**-**	3
3	Liu [30]	**★**	**-**	**-**	**-**	**★**	**-**	**★**	**-**	3
4	Santos [31]	**★**	**-**	**-**	**-**	**★**	**-**	**★**	**-**	3
5	Al Amri [32]	**★**	**★**	**-**	**-**	**★**	**-**	**★**	**-**	4
6	Murugesan [33]	**★**	**★**	**-**	**-**	**★**	**-**	**★**	**-**	4
7	Vargas [34]	**★**	**★**	**-**	**-**	**★**	**-**	**★**	**-**	4
8	Du [35]	**★**	**★**	**★**	**★**	**★**	**★**	**★**	**★**	8
9	Matias [12]	**★**	**★**	**-**	**-**	**★**	**-**	**★**	**-**	4
10	Arango [36]	**★**	**★**	**-**	**-**	**★**	**-**	**★**	**★**	5
11	Lima [37]	**★**	**★**	**-**	**-**	**★**	**-**	**★**	**★**	5
12	Song [38]	**★**	**★**	**-**	**-**	**★**	**-**	**★**	**★**	5
13	Tavares [39]	**★**	**★**	**-**	**-**	**★**	**-**	**★**	**-**	4
14	Gibas-Stanek [40]	**★**	**★**	**-**	**-**	**★**	**-**	**★**	**-**	4

Star (★) = item present.

## Data Availability

The data presented in this study are available on request from the corresponding author. The data are not publicly available due to privacy restrictions.

## Abbreviations

AC	alveolar crest
CBCT	cone-beam computed tomography
CEJ	cemento-enamel junction
CSS	cross-sectional study
CT	computed tomography
F	female
FOV	field of view
FP	Frankfurt plane
IZC	infrazygomatic crest
M	male
MBS	mandibular buccal shelf
OP	occlusal plane
TAD	temporary anchorage device = miniscrew
ZP	zygomatic process
5–6	region between maxillary second premolar and first molar
6D	distobuccal root of first maxillary molar
6IR	middle of the buccal furcation of the maxillary first molar (equal distances to mesial and distal buccal root)
6M	mesiobuccal root of maxillary first molar
6–7	region between maxillary first and second molar
7D	distobuccal root of maxillary second molar
7IR	middle of the buccal furcation of the maxillary second molar (equal distances to mesial and distal buccal root)
7M	mesiobuccal root of second maxillary molar
